# Prevalence and molecular characterization of *Dirofilaria immitis* in road killed canids of northern Iran

**DOI:** 10.1186/s12917-022-03270-z

**Published:** 2022-05-02

**Authors:** Meysam Sharifdini, Mahan Karimi, Keyhan Ashrafi, Mostafa Soleimani, Hamed Mirjalali

**Affiliations:** 1 Department of Medical Parasitology and Mycology, School of Medicine, Guilan University of Medical Sciences, Rasht, Iran; 2 Foodborne and Waterborne Diseases Research Center, Research Institute for Gastroenterology and Liver Diseases, Shahid Beheshti University of Medical Sciences, Tehran, Iran

**Keywords:** *Dirofilaria immitis*, Dogs, Jackals, Iran, PCR

## Abstract

**Background:**

*Dirofilaria immitis* is a mosquito-borne filarial nematode, which infects primarily wild and domestic canids, causing cardiopulmonary dirofilariasis. The aim of the present study was to determine the prevalence and characterize molecular features of *D. immitis* in road killed canids, northern Iran.

**Methods:**

The carcasses of 53 road killed canids including 18 dogs (*Canis familiaris*), and 35 golden jackals (*C. aureus*) were necropsied in both Mazanderan and Guilan provinces, northern Iran. The molecular analyses were conducted based on the cytochrome oxidase (*Cox*) 1 and 18S ribosomal RNA (rRNA) genes.

**Results:**

The heartworm infection was found in 55.6% of dogs and 22.9% of jackals. Our study revealed significantly higher prevalence of *D. immitis* in dogs compared to jackals (*P* = 0.031). The prevalence of *D. immitis* was no statistically significant between males and females in both dogs and jackal (*P* > 0.05). Comparison of the *Cox*1 gene sequences with available data in the GenBank illustrated 100% similarity with *D. immitis* isolates from different hosts in European, Asian, and South American continents. Moreover, the 18S rRNA gene sequences showed 100% identity with dog isolates from Japan and French Guiana.

**Conclusions:**

This study confirms the high prevalence of *D. immitis* in dogs and jackals of northern Iran. Developing control programs to prevent transmission of the disease is necessary for dogs and humans in the study areas.

## Background

*Dirofilaria immitis,* Leidy, 1856 (Spirurida: Onchocercidae), heartworm, is a mosquito-borne filarial nematode that infects wild and domestic canids, felids, and humans [1]. This nematode is transmitted by various mosquitoes belonging to the genera *Aedes*, *Armigeres*, *Culex*, *Anopheles*, and *Mansonia* [2, 3]. Adult worms of *D. immitis* often reside in the pulmonary arterial system and the heart of canids and felids. However, humans are less suitable hosts, in which the parasites usually do not mature sufficiently to produce microfilariae [1].

The main clinical symptoms of canine dirofilariasis include persistent cough, dyspnea, lethargy, weight loss, diminished exercise tolerance, coughing up blood, abdominal distention, and in advanced forms of the disease can lead to congestive heart failure, intravascular hemolysis, and pulmonary thromboembolism, which are often fatal if remain untreated [1, 4]. *D. immitis* infection in humans usually causes pulmonary nodules, and in fewer cases, ocular and subcutaneous involvements [1, 5–7]. *D. immitis* was detected in the heart and major blood vessels of humans in few cases, as well [8].

Diagnosis of *D. immitis* infection in canids is mostly based on microscopy to detect microfilariae in stained blood smear and modified Knott’s technique, but serological tests and molecular methods are the other employed methods [9, 10].

*D. immitis* is mostly found in subtropical and temperate areas in the world, where there is a population density of canine reservoirs and mosquito vectors [1, 11]. Several epidemiological studies have shown that the prevalence rates of dirofilariasis in dogs were 1.4 to 78.6% in different provinces of Iran [12–21]. There are also reports of *D. immitis* in jackals in some parts of Iran [22, 23]. In addition, rare cases of *D. immitis* infection have been reported in cats [24, 25]. Moreover, the prevalence of *D. immitis* infection of mosquitoes in Iran is high and *Cx. theileri* is the main vector of *D. immitis* in the country [3, 26].

Human dirofilariasis has a dramatically increasing trend in European and American countries [1, 27], and in past decades, several human cases of dirofilariasis have been reported in different parts of Iran [6, 7, 28–32]*.*

Recently, molecular methods are applied for reliable identification and phylogenetic analysis of canine filarial nematodes. Nevertheless, there are limited epidemiological and molecular information about *D. immitis* infection in canids especially jackals from the northern parts of the Iran, where environmental conditions are suitable for sustaining and expanding the infection. Therefore, the purpose of this study was to determine the prevalence of *D. immitis* obtained from road killed canids in northern Iran and investigate their genetic diversity using partial mitochondrial cytochrome oxidase subunit 1 (*Cox*1) and 18S ribosomal RNA (rRNA) genes.

## Methods

### Study area

**Fig. 1:**
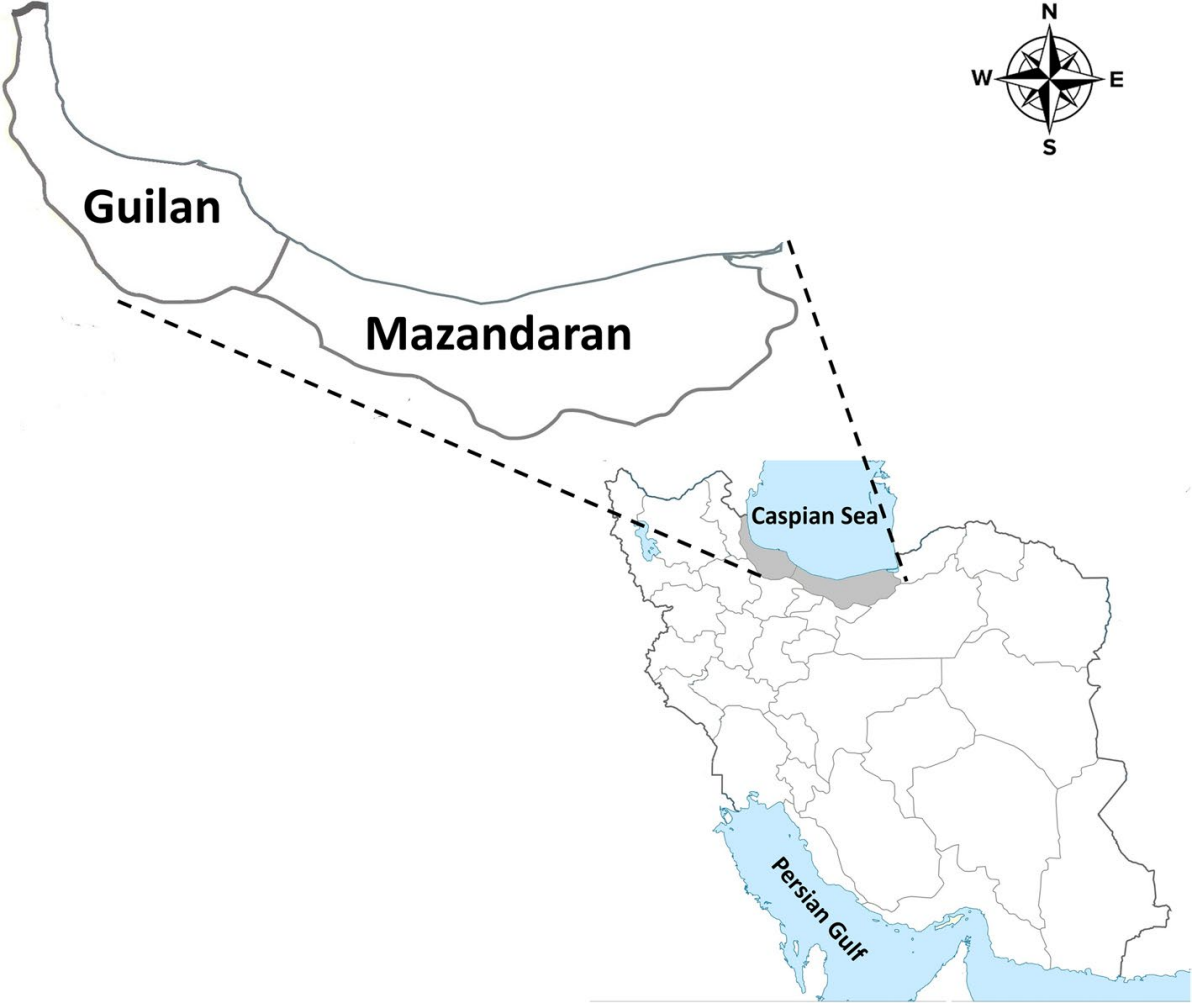
Map of Iran showing the geographical location of Guilan and Mazanderan Provinces in northern Iran

A cross-sectional study was conducted from October 2017 to September 2019, in both Guilan (37. 2774 N 49. 5890 E) and Mazanderan (36.5656 N, 53.0588 E) provinces, north of Iran. These provinces are located in southern border of the Caspian Sea and their southern parts are surrounded by Alborz mountains (Fig. 1). These provinces cover an area of 38,544 km^2^ and have humid subtropical climate with mean annual rainfall of 1359 mm for Guilan province and about 1000 mm for Mazanderan province. The average relative humidity is about 80%, which reaches a maximum in autumn and winter, and decreases in summer and spring [33, 34].

### Samples collection

In current study, a total of 53 carcasses of road killed canids including 18 dogs (*Canis familiaris*), and 35 golden jackals (*C. aureus*) were collected from both Mazanderan and Guilan provinces, northern Iran. The carcasses were examined for the presence of adult *D. immitis* worms in the heart and pulmonary artery. The obtained specimens were preserved in 70% ethanol for parasitological and molecular examinations. For determination of morphological characteristics, the nematodes were stained by lactophenol and azocarmine as a temporary mount [35].

### Molecular analysis

For DNA extraction, one worm of each infected canids (*n* = 18) was washed three times in distilled water to remove ethanol. Next, total genomic DNA was extracted using a commercial DNA extraction kit (Yekta Tajhiz Azma, Tehran, Iran), according to the manufacturer’s instructions and kept at − 20 °C until use. PCR reactions were performed in 30 μL volumes containing 2X red PCR premix (Ampliqon, Odense, Denmark), 20 pmol of each primer and 1 μL of extracted DNA. The forward primer COIintF (5′ -TGATTGGTGGTTTTGGTAA-3′) and the reverse primer COIintR (5′-ATAAGTACGAGTATCAATATC-3′) were used to amplify an about 689 bp fragment of the *Cox*1 gene [36]. Also, a 1155-bp long amplicon of 18S rRNA gene was amplified using the forward primer Fwd.18S.631 (5ʹ- TCGTCATTGCTGCGGTTAAA-3ʹ) and the reverse primer Rwd.18S.1825r (5ʹ- GGTTCAAGCCACTGCGATTAA-3ʹ) [37]. PCR conditions for *Cox*1 gene were an initial denaturing step of 94 °C for 5 min and 35 cycles followed by denaturing step at 94 °C for 30 s, annealing step of 52 °C for 45 s, and 60 s of extension at 72 °C, and 72 °C for 7 min as a final extension. The thermal PCR profiles for 18S rRNA gene consisted of initial denaturation at 95 °C for 6 min followed by 30 cycles of 95 °C for 60 s (denaturation), 58 °C for 20 s (annealing), and at 72 °C for 45 s (extension) with a final extension of 72 °C for 10 min. The PCR products were separated by electrophoresis on a 1.5% agarose gel and visualized using a UV transilluminator (UVITEC, Cambridge, UK). The amplification products were sent to a domestic sequencing company (Codon genetic company, Tehran, Iran) for sequence determination using the Sanger method.

### Phylogenetic analysis

The sequence results were edited and trimmed using Chromas version 2.01 (Technelysium Pty Ltd., Brisbane, Queensland, Australia) and compared to the GenBank database using the BLAST programs (http://www.ncbi.nlm.nih.gov/). The sequences of 18S rRNA and *Cox*1 genes were submitted to the GenBank database (Accession Numbers: MZ266347- MZ266364 for *Cox1* and MZ265267-MZ265284 for 18S rRNA gene). Phylogenetic analysis was performed with sequences obtained in the present study along with the reference sequences, which were deposited in the GenBank database, using Maximum-Likelihood algorithm and Tamura-3-parameter model in the MEGA 6.0 software. The reliability of the phylogenetic trees was supported with bootstrap value based on 1000 replications.

## Results

**Fig. 2:**
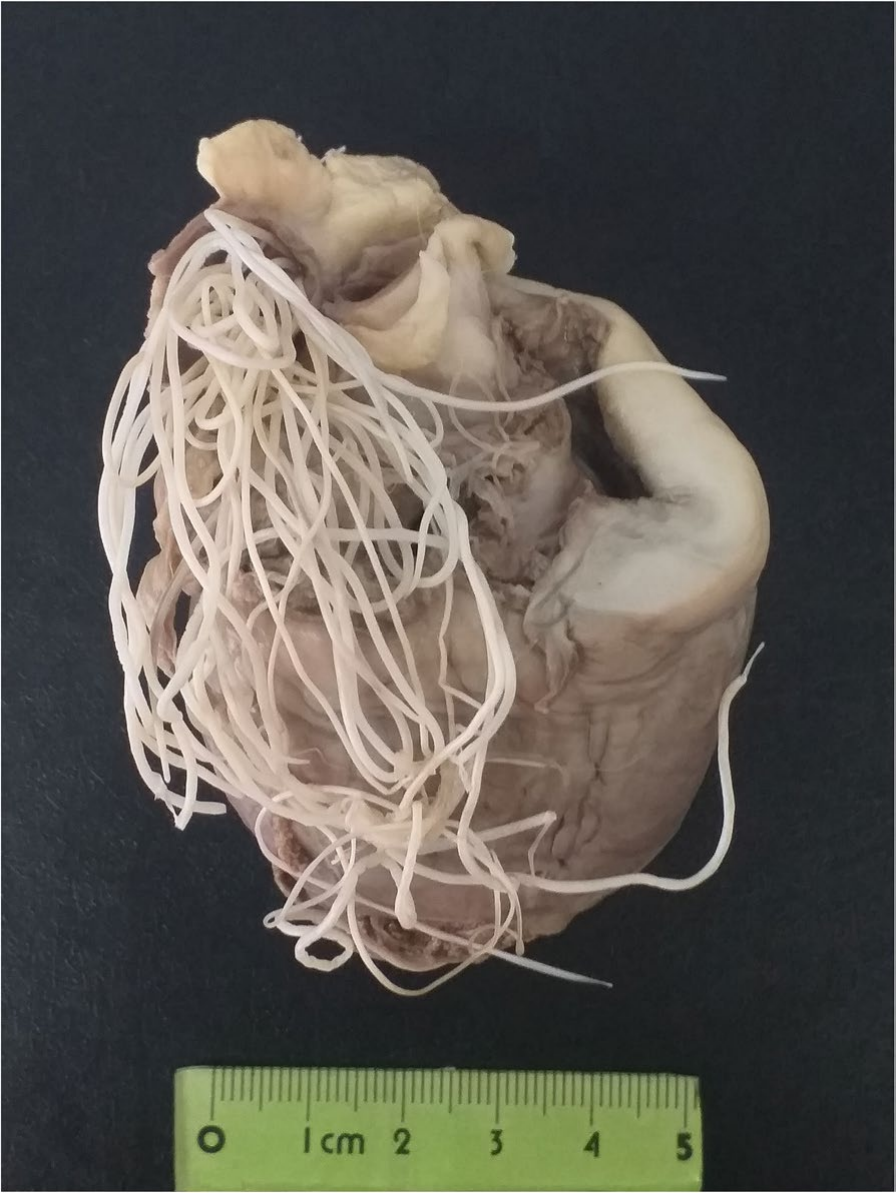
Numerous adult worms *Dirofilaria immitis* in a dog heart

Out of 53 carcasses of examined road killed canids, *D. immitis* was found in 10 of dogs (55.6%) and eight of jackals (22.9%) (Fig. 2). There were significant differences between the infection with *D. immitis* and types of canids (*P* = 0.031). The rate of infectivity in male and female dogs was 53.8% (7/13) and 60% (3/5), respectively, and was no statistically significant (*P* > 0.05). In addition, in jackals, 20% (4/20) of the males and 26.7% (4/15) of the females were infected with the *D. immitis* and no statistically significant difference was seen between the infection and the gender (*P* > 0.05). Only three (8.57%) of 35 jackals were puppies and the remaining 32 (91.43%) were adults. All of 18 dogs were adults, as well. One of three jackal puppies and seven out of 32 adult jackals were infected with heartworm (Table 1).

**Table 1:** Frequency and prevalence of *Dirofilaria immitis* in road killed canids from northern Iran according to the sex and age class

Host	Age class		Sex		Total (*n* = 53) No. (%)
	Pup No. (%)	Adult No. (%)	Male No. (%)	Female No. %	
*Canis aureus* (*n* = 35)	1 (33.3)	7 (21.9)	4 (20)	4 (26.7)	8 (22.9)
*Canis familiaris* (*n* = 18)	0 (0)	10 (100)	7 (53.8)	3 (60)	10 (55.6%)

In Guilan province, 75% (9/12) of dogs and 35% (7/20) of jackals were infected with *D. immitis*. In Mazanderan province, the infection rate in dogs and jackals was 16.7% (1/6) and 6.7% (1/15), respectively. A statistically significant difference was found between the two provinces regarding infection rates of dogs (*P* = 0.043); but not jackals (*P* > 0.05).

**Fig. 3:**
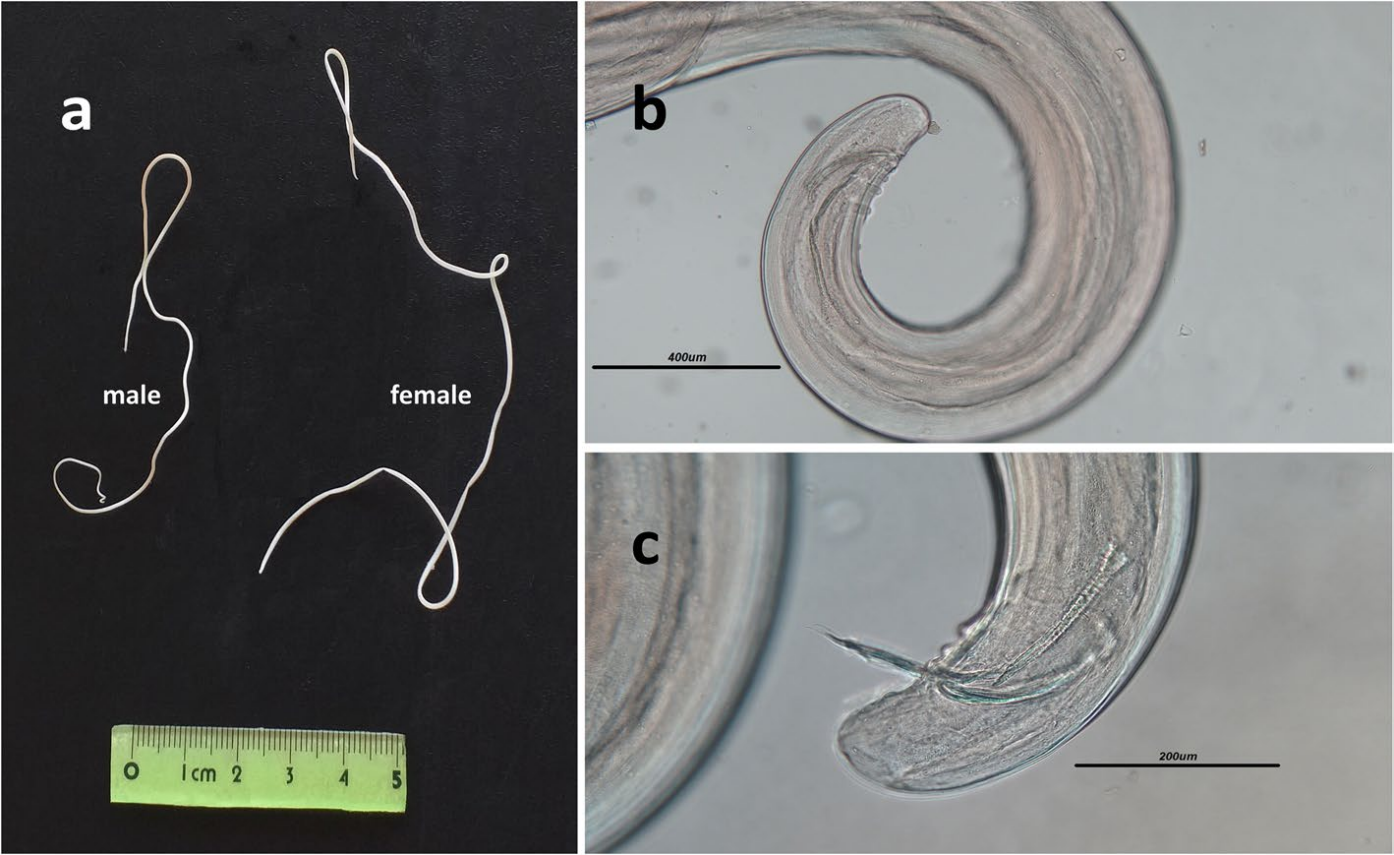
Male and female adult worms of *Dirofilaria immitis* (**a**) and posterior end of a male *Dirofilaria immitis* showing spicules and pre-anal papillae (**b** and **c**)

Male and female heartworms were identified based on the body length and morphological characteristics. Male worms are shorter than female and their coiled posterior end shows the presence unequal spicules and pre-anal papillae, but the females are larger and straight on both ends (Fig. 3). The range in total worm burden was 2–105 with a mean 23.5 for infected dogs and 1–23 with a mean 6.62 for jackals.

The genetic divergence within the specimens of *D. immitis* obtained from this study was 0% based on the partial *Cox*1 and 18S rRNA genes. The intra-species distance rate within our sequences of *D. immitis* and those available in the GenBank amounted to 0–0.7% and 0–2.9% for *Cox*1 and 18S rDNA fragments, respectively. Inter-generic differences based on the partial *Cox*1 gene between our sequences of *D. immitis* with *D. repens,* Railliet & Henry, 1911 (Spirurida: Onchocercidae), *Acanthocheilonema viteae,* Krepkogorskaja, 1933 (Spirurida: Onchocercidae), *Loa loa,* Cobbold, 1864 (Spirurida: Onchocercidae), *Brugia malayi,* Brug, 1927 (Spirurida: Onchocercidae), and *Wuchereria bancrofti,* Seurat, 1921 (Spirurida: Onchocercidae) were 10–10.3%, 16%, 12.5%, 17.1% and 15.2%, respectively. Also, the nucleotide divergence based on the partial 18S rRNA gene between our sequences of *D. immitis* with *D. repens*, *B. malayi*, *W. bancrofti, L. loa*, *Onchocerca cervicalis,* Railliet & Henry, 1910 (Spirurida: Onchocercidae), *A. viteae,* and *A. reconditum,* Grassi, 1889 (Spirurida: Onchocercidae) was 3–3.3%, 3.1%, 3%, 3.1%, 3.1%, 3.6% and 3.7%, respectively.

**Fig. 4:**
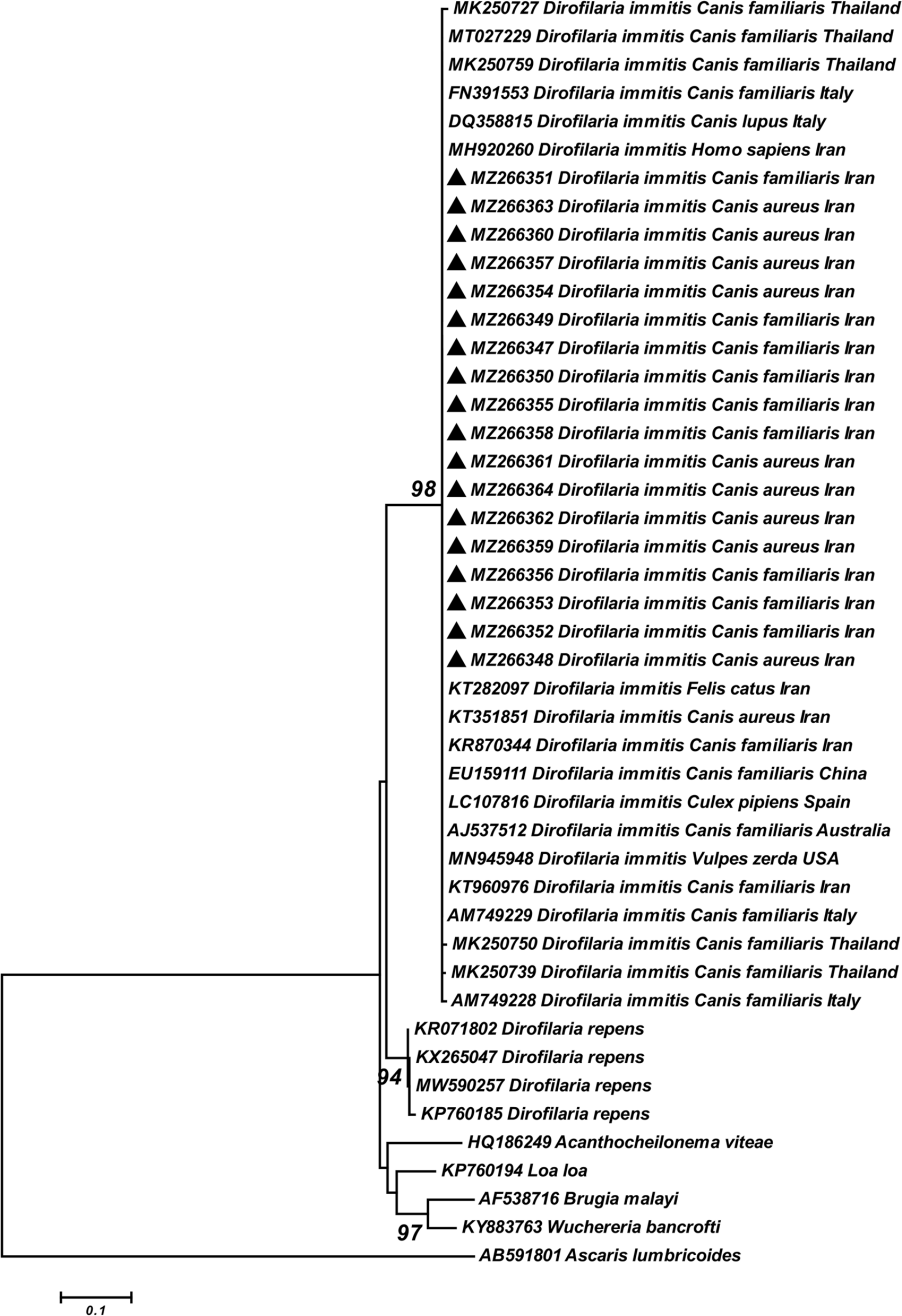
Phylogenetic tree of *Cox1* gene sequences of the *Dirofilaria immitis* obtained in this study (▲) and reference sequences retrieved from the GenBank. The tree was constructed based on the maximum likelihood method and the Tamura 3-parameter model in MEGA6. *Ascaris lumbricoides* sequence was used as the out group. Bootstrap values lower than 50 were omitted

The BLAST analysis based on the partial sequences of the *Cox*1 gene indicated our sequences of *D. immitis,* obtained from jackals and dogs (MZ266347-MZ266364), presented 100% homology with *D. immitis* isolated from dogs (KR870344 and KT960976), cat (KT282097), jackal (KT351851), and human (MH920260) in Iran, dog isolates of *D. immitis* from Thailand (MK250759 and MT027229), Italy (FN391553 and AM749229), China (EU159111) and Australia (AJ537512), jackal isolate from Italy (DQ358815), fennec fox isolate (MN945948) from USA and also *Culex pipiens* Linnaeus, 1758 (Diptera: Culicidae) isolate (LC107816) from Spain. In addition, our sequences had 99.3–99.6% and 99.5% similarity with dog isolates of *D. immitis* from Thailand (MK250727, MK250739 and MK250750) and Italy (AM749228), respectively. The phylogenetic construction based on the *Cox*1 gene illustrated that all *D. immitis* isolates obtained in this study placed along with other isolates of *D. immitis* from different hosts and countries in one major clade with high statistical support (Fig. 4). This major clade was located as monophyletic and a sister taxon of *D. repens* isolates.

**Fig. 5:**
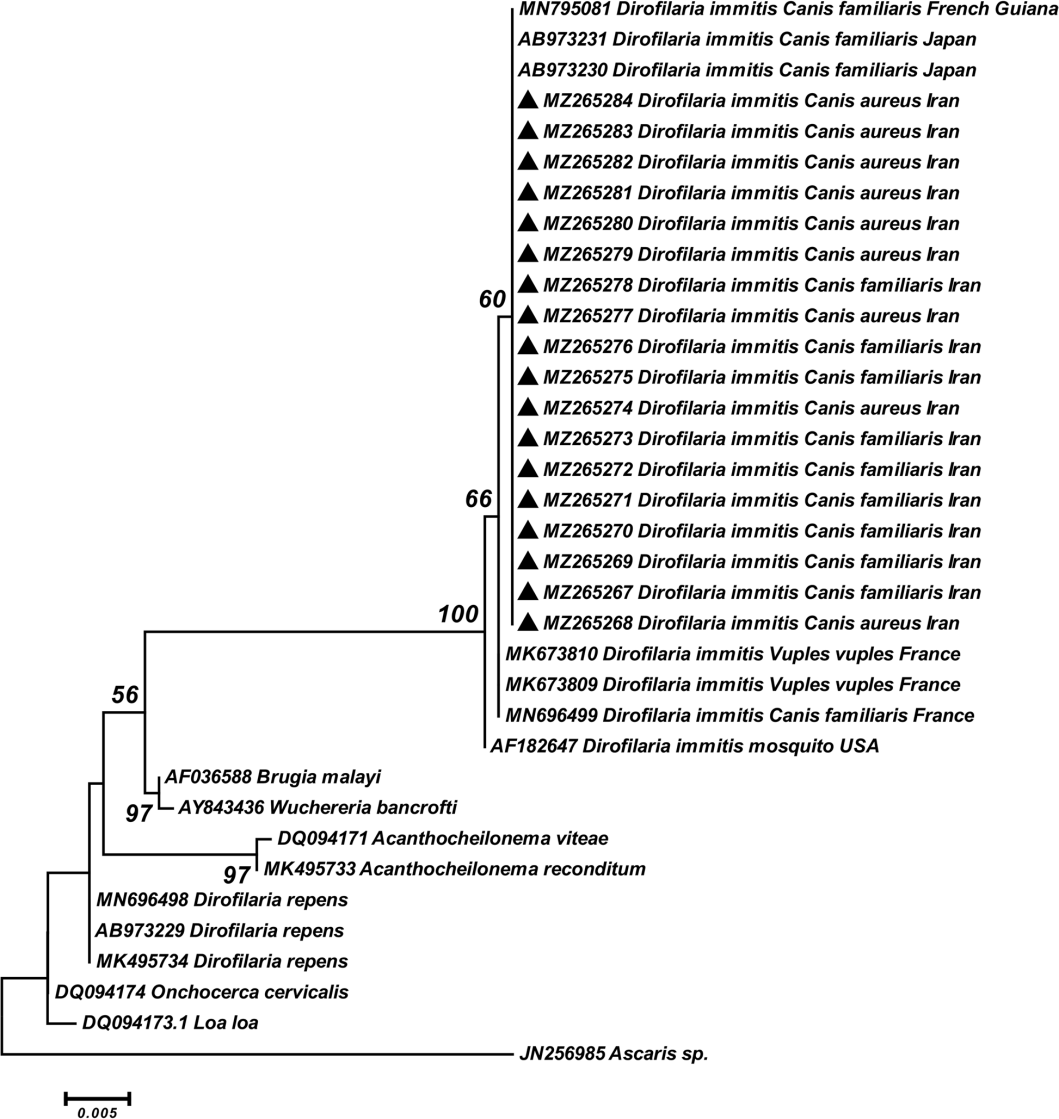
Phylogenetic tree of 18S rRNA gene sequences of the *Dirofilaria immitis* obtained in this study (▲) and reference sequences retrieved from GenBank. The tree was constructed based on the maximum likelihood method and the Tamura 3-parameter model in MEGA6. *Ascaris* sp*.* sequence was used as the out group. Bootstrap values lower than 50 were omitted

The BLAST analysis based on 18S rRNA gene showed that the *D. immitis* sequences obtained in this study (MZ265267-MZ265284) had 100% similarity with *D. immitis* isolated from dogs in Japan (AB973230 and AB973231) and French Guiana (MN795081). Also, the sequences presented 99.9% with dog (MK673810) and re fox (MK673809 and MK673810) isolates of *D. immitis* from France. Additionally, our sequences had 97.1% identity with mosquito isolate of *D. immitis* (AF182647) from USA. According to phylogenetic analysis based on the 18S rRNA gene (Fig. 5), our sequences were grouped in one cluster with *D. immitis* isolates obtained from dogs in Japan and French Guiana. In addition, dog and re fox isolates from France were placed close to the mentioned clade. Meanwhile, the mosquito isolate of *D. immitis* from USA was located separately from other isolates of *D. immitis*.

## Discussion

*D. immitis* has a cosmopolitan distribution and infects dogs, cats, and wild canids, as well as humans [1]. Canine dirofilariasis has previously been reported from different parts of Iran with variable rate of infections [12, 38, 39], and also human infections associated with this species have been sporadically reported from the same regions that canine infections have been reported [6, 28, 38].

This study updates our understanding about the situation of *D. immitis* infection in dogs and jackals in northern Iran and provides new molecular data about this nematode based on both *Cox1* and 18S rRNA genes in the study area. In the present study, 55.6% of dogs were found to be infected with adult heartworm. The prevalence rates for *D. immitis* in dogs of different regions of Iran are related to the environmental and ecological features, with the highest prevalence reported from Guilan (78.6%) and Mazandaran (50%) provinces in the southern coasts of the Caspian Sea [21]. These provinces have mean annual temperatures in optimum range for mosquito development. Also, the mean annual humidity and rainfall of both Guilan and Mazandaran provinces is high which affect the propagation of the vectors [16]. The low prevalence was recorded in some reigns such as Esfahan (0.9%) [21], Tehran (1.4%) [17], Lorestan (6.9%) [21], Kermanshah (18.3%) [13], and Tabriz (11.6%) [40] could be due to unfavorable temperature for mosquito activity and decrease in yearly rainfall. Heartworm infection in dogs is widespread in many parts of the world, and the prevalence rate based on continent was Australia 22.68%, Asia 12.07%, America 11.60%, Europe 10.45% and Africa 7.57% [41]. The prevalence of *D. immitis* varies among different regions of the world due to some epidemiological factors, such as the distribution of the mosquito species, mosquito population density, environmental temperature, dog population density, and age of host [41].

In this study, *D. immitis* was detected in 22.9% of road killed jackals. This parasite has previously been reported in 8.9% (4/45) golden jackals from North Khorasan, northeast Iran [23]. In a similar study, the adult heartworm was found in one of eleven road killed jackals (9%) in Guilan province, northern Iran [22]. The existence of *D. immitis* in jackals (*Canis aureus*) has been reported in some European countries [4, 42]. The heartworm infection was found in jackals with a prevalence of 8.9% in Bulgaria [4]. In Serbia, adult *D. immitis* was also detected in 7.32% of jackals [42]. This wildlife reservoir might be a potential source of infection for dogs and humans in the endemic areas.

The present study revealed that *D. immitis* in dogs was significantly higher than jackals. Similar to our finding, Vafae Eslahi et al. (2017) showed that heartworm infection in dogs (25.9%) is higher than golden jackals (9%) in northern Iran [22]. However, in other study in northeast Iran, *D. immitis* infection in jackals (8.9%) was much higher than dogs (0%), and foxes (0%) [23]. Likewise, the heartworm infection was reported with higher prevalence in jackals (7.32%) than other wild carnivores including red foxes (1.55%) and wolves (1.43%) in Serbia [42].

The results of this study showed that there was no statistically significant difference between infectivity with *D. immitis* and sex in both dogs and jackals. Similar findings were indicated in most other studies on dogs [12, 13, 43–46] and jackals [23]. Meanwhile, a few studies reported that the infectivity rate in males was higher than female dogs and jackals [42, 47].

In the present study, *D. immitis* in dogs was significantly higher found in Guilan province. Also, the infection rate in jackals of Guilan province was more than Mazandaran; however, the difference was not statistically significant. Similar to our results, other studies also reported a significantly higher rate of heartworm infection in dogs in Guilan province than Mazandaran province, both located in the southern coasts of the Caspian sea [16, 21]. Although, the mean annual rainfall of Guilan province is a little more than Mazandaran, but it is necessary to do more investigations on other differences among these provinces.

In last few decades, DNA sequence-based methods have been widely applied as useful tools to assess genetic variations, identification, classification, and phylogenetic analysis of filarial nematodes [37, 43, 48, 49]. In the current study, no generic differences were found between *D. immitis* isolates obtained from dogs and jackals based on the *Cox*1 and 18S rRNA genes. Comparisons of our sequences and those available in the GenBank showed that the interspecific variation of the 18S rRNA gene (0–2.9%) is higher than *Cox*1 gene (0–0.7%).

The phylogenetic tree based on the *Cox*1 gene illustrated that our samples clustered with the major *D. immitis* group consisting of isolates from different hosts and geographically areas in European, Asian, and South American continents. Our *Cox*1 sequences had 100% similarity with most previously reported *D. immitis* sequences from Iran and other countries including Thailand, Italy, China, Australia, Spain, and the USA. Within this major clade, only four isolates form Thailand and Italy exhibited 0.4–0.7% differences to our sequences. This finding along with other studies [23, 48] indicate a low genetic variability based on the *Cox*1 gene among *D. immitis* isolates in various geographic locations*.*

Available 18S rRNA sequences of *D. immitis* in the GenBank are low; however, the phylogenetic analysis showed that our sequences were grouped with 100% identity with dog isolates from Japan and French Guiana. Moreover, the pairwise homology analysis revealed a single nucleotide polymorphism (SNP) (T/C) in dog and fox isolates from France, which was responsible for separating them in the tree.

## Conclusions

The results of the current study confirm high prevalence of *D. immitis* in dogs and jackals in northern Iran. Consequently, the transmission risk of the filarial nematode with zoonotic potential must be informed to physicians and veterinarians in the regions. Hence, it is recommended that an accurate and consistent screening and treatment program should be conducted among dogs in the study area. Our study provided the genetic structure of *D. immitis* based on both *Cox*1 and 18S rRNA genes, with no polymorphism found among the two canids in the different geographic locations. Providing additional genetic markers as well as obtaining more isolates from various geographical region would be useful for gaining a better understanding of genetic variation among populations of *D. immitis*.

## Data Availability

All data generated or analyzed during this study have been included in this published article. The sequence data were deposited in the GenBank database under the accession numbers: MZ266347- MZ266364 for *Cox1* and MZ265267-MZ265284 for 18S rRNA gene.

## Abbreviations

18S rRNA: 18S ribosomal RNA
*Cox1*: Cytochrome oxidase 1
PCR: Polymerase chain reaction

## References

[1] Simón F, Siles-Lucas M, Morchón R, González-Miguel J, Mellado I, Carretón E (2012). Human and animal dirofilariasis: the emergence of a zoonotic mosaic. Clin Microbiol Rev.

[2] Shaikevich E, Bogacheva A, Ganushkina L.* Dirofilaria* and* Wolbachia* in mosquitoes (Diptera: Culicidae) in central European Russia and on the Black Sea coast. Parasite. 2019;26:2. 10.1051/parasite/2019002 PMC6333102 30644356

[3] Riahi SM, Yusuf MA, Azari-Hamidian S, Solgi R. Prevalence of* Dirofilaria immitis* in mosquitoes (Diptera)–systematic review and meta-analysis. J Nematol. 2021;53:e2021–12. 10.21307/jofnem-2021-012 PMC8039976 33860239

[4] McCall JW, Genchi C, Kramer LH, Guerrero J, Venco L (2008). Heartworm disease in animals and humans. Adv Parasitol.

[5] Falidas E, Gourgiotis S, Ivopoulou O, Koutsogiannis I, Oikonomou C, Vlachos K, et al. Human subcutaneous dirofilariasis caused by *Dirofilaria immitis* in a Greek adult. J Infect Public Health. 2016;9:102–4. 10.1016/j.jiph.2015.06.005 26166816

[6] Mirahmadi H, Maleki A, Hasanzadeh R, Ahoo MB, Mobedi I, Rostami A. Ocular dirofilariasis by* Dirofilaria immitis* in a child in Iran: a case report and review of the literature. Parasitol Int. 2017;66:978–81. 10.1016/j.parint.2016.10.022 27815230

[7] Parsa R, Sedighi A, Sharifi I, Bamorovat M, Nasibi S. Molecular characterization of ocular dirofilariasis: a case report of *Dirofilaria immitis* in south-eastern Iran. BMC Infect Dis. 2020;20:1–5. 10.1186/s12879-020-05182-5 PMC7367333 32677898

[8] Theis J (2005). Public health aspects of dirofilariasis in the United States. Vet Parasitol.

[9] Lee S-E, Song K-H, Liu J, Kim M-C, Park B-K, Cho K-W, et al. Comparison of the acid-phosphatase staining and polymerase chain reaction for detection of *Dirofilaria repens* infection in dogs in Korea. J Vet Med Sci. 2004;66:1087–9. 10.1292/jvms.66.1087 15472472

[10] Solgi R, Sadjjadi SM, Mohebali M, Zarei Z, Golkar M, Raz A. Development of new recombinant DgK antigen for diagnosis of *Dirofilaria immitis* infections in dogs using ELISA technique and its comparison to molecular methods. Iran Biomed J. 2018;22:283. 10.22034/ibj.22.4.283 PMC5949131 29031244

[11] Otranto D, Dantas-Torres F, Brianti E, Traversa D, Petrić D, Genchi C (2013). Vector-borne helminths of dogs and humans in Europe. Parasites Vectors.

[12] Ranjbar-Bahadori S, Veshgini A, Shirani D, Eslami A, Mohieddin H, Shemshadi B (2011). Epidemiological aspects of canine dirofilariasis in the north of Iran. Iran J Parasitol.

[13] Bohloli Oskoii S, Sadeghi E, Hashemian A, Ghaffari KS (2013). Study on Shepherd dog dirofilariosis in Kermanshah province in 2011–2012. J Vet Lab Res.

[14] Bokaie S, Mobed I, Mohebali M, Hoseini S, Nadim A (1998). A study of dirofilariasis prevalence in dogs in Meshkin-Shahr area. northwest Iran. J Fac Vet Med..

[15] Jafari S, Gaur S, Khaksar Z. Prevalence of *Dirofilaria immitis* in dogs of Fars province of Iran. J Appl Anim Res. 1996;9:27–31.

[16] Malmasi A, Hosseini S, Aramoon M, Bahonar A, Seifi HA. Survey of canine *Dirofilaria immitis* infection in Caspian provinces of Iran. Iran J Vet Res. 2011;12:340–4.

[17] Meshgi B, Eslami A (2001). Study on filariasis of sheepdogs around of Tehran. J Fac Vet Med Tehran Univ.

[18] Meshgi B, Eslami A, Ashrafi HJ (2002). Epidemiological survey of blood filariae in rural and urban dogs of Tabriz. J Fac Vet Med Tehran Univ.

[19] Ranjbar BS, Hekmat KA (2007). A study on filariosis of stray dogs in Garmsar. J Vet Res.

[20] Ranjbar BS, Mohtasham R, Eslami A, Meshki B (2005). Study on blood filariosis of dog in Tonekabon 2005. J Vet Res.

[21] Hosseini SH, Manshori-Ghaishghorshagh F, Ramezani M, Nayebzadeh H, Ahoo MB, Eslamian A (2022). Canine microfilaraemia in some regions of Iran. Parasit Vectors.

[22] Eslahi AV, Eshrat Beigom K, Mobedi I, Sharifdini M, Badri M, Mowlavi G (2017). Road killed carnivores illustrate the status of zoonotic helminthes in Caspian Sea littoral of Iran. Iran J Parasitol.

[23] Heidari Z, Kia EB, Arzamani K, Sharifdini M, Mobedi I, Zarei Z, et al. Morphological and molecular identification of *Dirofilaria immitis* from Jackal (*Canis aureus*) in North Khorasan, northeast Iran. J Vector Borne Dis. 2015;52:329–33. 26714514

[24] Khodabakhsh M, Malmasi A, Mohebali M, Zarei Z, Kia EB, Azarm A. Feline dirofilariosis due to *Dirofilaria immitis *in Meshkin Shahr district. Northwestern Iran Iran J Parasitol. 2016;11:269–73. PMC5236107 28096864

[25] Alborzi A, Mosallanejad B, Najafabadi MG, Nikpoor Z. Infestation of heartworm (*Dirofilaria immitis*) in a cat in Ahvaz City: a case report. J Vet Res. 2010;65:255–71.

[26] Solgi R, Sadjjadi SM, Mohebali M, Djadid ND, Raz A, Zakeri S, et al. Susceptibility of *Anopheles stephensi* (Diptera: Culicidae) to *Dirofilaria immitis* (Spirurida: Onchocercidae). Russ J Nematol. 2017;25:121–7.

[27] Simón F, López-Belmonte J, Marcos-Atxutegi C, Morchón R, Martín-Pacho J (2005). What is happening outside North America regarding human dirofilariasis?. Vet Parasitol.

[28] Jamshidi A, Jamshidi M, Mobedi I, Khosroara M (2008). Periocular dirofilariasis in a young woman: a case report. Korean J Parasitol.

[29] Tafti MF, Hajilary A, Siatiri H, Rokni M, Mobedi I, Mowlavi G (2010). Ocular dirofilariasis, a case report. Iran J Parasitol.

[30] Ashrafi K, Golchai J, Geranmayeh S. Human subcutaneous dirofilariasis due to* Dirofilaria* (*Nochtiella*) *repens*: clinically suspected as cutaneous fascioliasis. Iran J Public Health. 2010;39:105–9. PMC3468962 23112998

[31] Tavakolizadeh S, Mobedi I (2009). Orbital dirofilariasis in Iran: a case report. Korean J Parasitol.

[32] Maraghi S, Rahdar M, Akbari H, Radmanesh M, Saberi A. Human dirofilariasis due to *Dirofilaria repens* in Ahvaz-Iran: a report of three cases. Pak J Med Sci. 2006;22:211–3.

[33] Gholipoor Z, Khazan H, Azargashb E, Youssefi MR, Rostami A (2020). Prevalence and risk factors of intestinal parasite infections in Mazandaran province, North of Iran. Clin Epidemiology Glob Health.

[34] Rad LK, Mohammadi H (2015). Climate change assessment in Gilan province. Iran Int J Agric Crop Sci.

[35] Genchi C, Rinaldi L, Cringoli G. *Dirofilaria immitis* and *D. repens* in dog and cat and human infections. Mappe parassitologiche. 2007.

[36] Casiraghi M, Anderson T, Bandi C, Bazzocchi C, Genchi C. A phylogenetic analysis of filarial nematodes: comparison with the phylogeny of *Wolbachia* endosymbionts. Parasitology. 2001;122:93–103. 10.1017/s0031182000007149 11197770

[37] Laidoudi Y, Ringot D, Watier-Grillot S, Davoust B, Mediannikov O (2019). A cardiac and subcutaneous canine dirofilariosis outbreak in a kennel in central France. Parasite.

[38] Azari-Hamidian S, Yaghoobi-Ershadi M, Javadian E, Mobedi I, Abai M (2007). Review of dirofilariasis in Iran. J Guilan Univ Med Sci.

[39] Khedri J, Radfar MH, Borji H, Azizzadeh M, Akhtardanesh B (2014). Canine heartworm in southeastern of Iran with review of disease distribution. Iran J Parasitol.

[40] Varjoy MH, Helan JA, Salehi N, Bazmani A, Nematollahi A, Baran AI. Molecular detection and epidemiological aspects of *Dirofilaria immitis* in dogs in Tabriz and Suburbs. J Maz Univ Med Sci. 2016;26:20–31.

[41] Anvari D, Narouei E, Daryani A, Sarvi S, Moosazadeh M, Hezarjaribi HZ, et al. The global status of *Dirofilaria immitis* in dogs: a systematic review and meta-analysis based on published articles. Res Vet Sci. 2020;131:104–16. 10.1016/j.rvsc.2020.04.002 32330696

[42] Penezić A, Selaković S, Pavlović I, Ćirović D. First findings and prevalence of adult heartworms (*Dirofilaria immitis*) in wild carnivores from Serbia. Parasitol Res. 2014;113:3281–5.li. 10.1007/s00436-014-3991-9 24951168

[43] Zarei Z, Kia EB, Heidari Z, Mikaeili F, Mohebali M, Sharifdini M. Age and sex distribution of *Dirofilaria immitis* among dogs in Meshkin-Shahr, northwest Iran and molecular analysis of the isolates based on COX1 gene. Vet Res Forum. 2016;7:329–34. PMC5251356 28144425

[44] Liu C, Yang N, He J, Yang M, Sun M. Prevalence of *Dirofilaria immitis* in dogs in Shenyang. Northeastern China Korean J Parasitol. 2013;51:375–7. 10.3347/kjp.2013.51.3.375 PMC3712115 23864752

[45] Wang S, Zhang N, Zhang Z, Wang D, Yao Z, Zhang H, et al. Prevalence of *Dirofilaria immitis* infection in dogs in Henan province, central China. Parasite. 2016;23:43. 10.1051/parasite/2016054 PMC5782855 27739399

[46] Borthakur SK, Deka DK, Islam S, Sarma DK, Sarmah PC. Prevalence and molecular epidemiological data on *Dirofilaria immitis* in dogs from Northeastern States of India. Sci World J. 2015;2015:265385. 10.1155/2015/265385 PMC4320797 25685835

[47] Razi Jalali MH, Alborzi AR, Avizeh R, Mosallanejad B. A study on* Dirofilaria immitis* in healthy urban dogs from Ahvaz. Iran. 2010;11:357–62.

[48] Khanmohammadi M, Akhlaghi L, Razmjou E, Falak R, Emameh RZ, Mokhtarian K, et al. Morphological description, phylogenetic and molecular analysis of *Dirofilaria immitis* isolated from Dogs in the Northwest of Iran. Iran J Parasitol. 2020;15:57–66. PMC7244830 32489376

[49] Panayotova-Pencheva M, Šnábel V, Dakova V, Čabanová V, Cavallero S, Trifonova A, et al. *Dirofilaria immitis* in Bulgaria: the first genetic baseline data and an overview of the current status. Helminthologia. 2020;5(57):211–8. 10.2478/helm-2020-0026 PMC7425230 32855608

